# Pathogenesis of Hepatitis C During Pregnancy and Childhood

**DOI:** 10.3390/v4123531

**Published:** 2012-12-06

**Authors:** Armelle Le Campion, Ariane Larouche, Sébastien Fauteux-Daniel, Hugo Soudeyns

**Affiliations:** 1 Unité d’immunopathologie virale, Centre de recherche du CHU Sainte-Justine, 3175 Côte Sainte-Catherine, local 6735, Montreal, Quebec, H3T 1C5, Canada; E-Mails: a.le.campion@umontreal.ca (A.L.C); ariane.larouche@umontreal.ca (A.L.); fauteux.sebastien@gmail.com (S.F.-D.); 2 Department of Microbiology & Immunology, Faculty of Medicine, Université de Montréal, C.P. 6128, Succ. Centre-ville, Montreal, Quebec, H3C 3J7, Canada; 3 Department of Pediatrics, Faculty of Medicine, Université de Montréal, C.P. 6128, Succ. Centre-ville, Montreal, Quebec, H3C 3J7, Canada

**Keywords:** Hepatitis C virus, pathogenesis, mother-to-child transmission, pediatrics

## Abstract

The worldwide prevalence of HCV infection is between 1% and 8% in pregnant women and between 0.05% and 5% in children. Yet the pathogenesis of hepatitis C during pregnancy and in the neonatal period remains poorly understood. Mother-to-child transmission (MTCT), a leading cause of pediatric HCV infection, takes place at a rate of <10%. Factors that increase the risk of MTCT include high maternal HCV viral load and coinfection with HIV-1 but, intriguingly, not breastfeeding and mode of delivery. Pharmacological prevention of MTCT is not possible at the present time because both pegylated interferon alfa and ribavirin are contraindicated for use in pregnancy and during the neonatal period. However, this may change with the recent introduction of direct acting antiviral agents. This review summarizes what is currently known about HCV infection during pregnancy and childhood. Particular emphasis is placed on how pregnancy-associated immune modulation may influence the progression of HCV disease and impact MTCT, and on the differential evolution of perinatally acquired HCV infection in children. Taken together, these developments provide insights into the pathogenesis of hepatitis C and may inform strategies to prevent the transmission of HCV from mother to child.

## 1. Introduction

Hepatitis C virus (HCV) infection affects 130 to 170 million people worldwide, which accounts for 2 to 3% of the world's population [1]. This figure includes women of reproductive age and children. The prevalence of HCV infection in pregnant women is between 1 to 2% in the United States and Europe but may be as high as 8% in some developing countries [2,3,4]. HCV is transmitted via parenteral exposure to infected blood or contaminated materials, commonly in the context of injection drug use, and, less commonly, following sexual contacts with HCV-infected partners [5,6,7]. Mother-to-child transmission (MTCT) of HCV has been clearly documented, with reported rates averaging 5-10% [8,9,10,11,12,13,14,15,16]. Before 1992, pediatric HCV infection was predominantly associated with iatrogenic transmission. That changed following the implementation of universal screening of blood and blood products, when MTCT became the leading source of HCV acquisition in children. Unfortunately, to this day, most cases of hepatitis C in children from resource-limited settings can be traced to transfusion of inadequately-screened blood products and/or parenteral transmission [17,18]. Consistent with this, the prevalence of pediatric HCV infection ranges from 0.05% to 0.36% in developed countries and between 1.8% and 5% in the developing world [19,20]. 

The pathogenesis of HCV during pregnancy and the neonatal period remains poorly understood. During pregnancy, the maternal immune system must at the same time develop tolerance to paternal alloantigens to prevent maternal immune aggression against the foetus and maintain active immunity against HCV to protect both mother and foetus from the infection. Furthermore, this modulation of immune responses differs between the different stages of pregnancy [21]. How does pregnancy-associated immune modulation influence the progression of HCV infection and impact MTCT? This review will summarize what is currently known about HCV-specific immune responses on the maternal side, at the materno-foetal interface, and on the foetal side. Understanding how mother and foetus face up to HCV may provide some perspectives on possible clinical intervention as new antiviral treatments for hepatitis C, including direct-acting antiviral agents (DAAs), are emerging.

## 2. Hepatitis C during Pregnancy

Although HCV affects a significant number of women of reproductive age, few studies have actually examined the impact of chronic HCV infection on pregnancy outcomes. A report on 506 HCV-infected pregnant women in the United States showed a higher risk for premature rupture of membranes and, in women with excessive weight gain, a greater risk of developing gestational diabetes mellitus [22]. Increased prevalence of gestational diabetes was also found in pregnant, HCV-infected women from the Hepatitis C Vertical Transmission Study Group (n = 148) [23], and in a group of 555 HCV-infected women from the National Inpatient Sample [24]. Furthermore, the incidence of cholestasis of pregnancy is increased in women who are HCV antibody-positive, and this condition appears earlier during gestation compared to HCV-negative women [25,26]. A large population-based study that involved 749 HCV or hepatitis B virus (HBV)-seropositive women who delivered between 1988 and 2007 revealed significantly higher rates of preterm delivery, premature rupture of membranes, placental abruption, low birth weight, low Apgar scores at 1 min, congenital malformations, and overall perinatal mortality [27]. Increased risks for obstetric complications associated with chronic HCV infection were not observed in other studies, though these were generally limited by small sample sizes [28,29].

A series of seminal studies have shown that there is a decrease, and even normalization, of levels of serum alanine aminotransferase (ALT), a marker of liver inflammation and hepatocellular injury, during the second and third trimesters of pregnancy. Concomitant with the decrease in serum ALT, HCV viral load increases, reaching a peak during the third trimester [8,30,31]. Conversely, exacerbation of chronic hepatitis C, including significant rebound of ALT levels and significantly worsening liver histopathology (Knodell score; portal necrosis; lobular degeneration; inflammation), were reported in the post-partum period, along with a reduction in HCV plasma viral load [29,32,33,34,35,36]. Taken together, these observations led to the initial suggestion that pregnancy can worsen HCV-associated liver injury and that pregnancy-associated immune modulation can influence the course of HCV disease.

For decades, pregnancy in immunological terms was widely thought of as a type of allogeneic organ transplant, though one that did not lead to rejection by the maternal immune system. This conceptualization of immunology of pregnancy also led to the generalized assessment that gestation was a state of «immunologic weakness» where the pregnant woman exhibited increased susceptibility to infectious disease [37]. During pregnancy, the maternal immune system is exposed to paternal alloantigens and establishment of maternofoetal tolerance is required to sustain foetal integrity. In addition to several locally-acting mechanisms, systemic changes in the maternal immune system take place in order to facilitate tolerance of the foetus [38]. Indeed, pregnancy is associated with quantitative and qualitative modulation of maternal immunity [39,40]: immunoglobulin synthesis is increased [41] while T cell-mediated immune responses are repressed [42]. Oestrogen was shown to inactivate the intrathymic T-cell differentiation pathway in mice while at the same time activating an extrathymic differentiation pathway in the liver [43], a mechanism that was also observed to take place during normal pregnancy [44]. Regulatory T cells (Treg) were shown to play a crucial role in maternofoetal tolerance, and studies in humans have demonstrated an expansion of CD4^+^ CD25^+^ Treg cells beginning early in gestation and reaching a peak in the second trimester [45,46,47]. These findings are consistent with decreased resistance to intracellular pathogens and the remission of T cell-associated autoimmune diseases such as multiple sclerosis and rheumatoid arthritis, which is commonly observed during pregnancy [48,49,50]. Thus, the decrease in ALT levels and increased HCV viral load observed in the third trimester of pregnancy in women chronically infected with HCV could conceivably be explained by a pregnancy-associated decline in immune-mediated hepatocellular destruction [8,30].

However, in evolutionary terms, pregnancy represents one of the most critical periods for the continued existence of a given species [51]. During this period, the maternal immune system is essential to protect the mother from pathogens and prevent damage to the unborn child. Pregnant mice are capable of developing antiviral memory CD8^+^ T cell responses just like their non-pregnant littermates, and the numbers, proliferative capacity, and functional properties of these cells remain essentially intact [52,53]. We and others have shown that pregnant women infected with HCV were capable of mounting both humoral and cell-mediated immune responses directed against HCV antigens, including E2-p7-NS2-NS3 and HCV alternate reading frame protein (ARFP), and that these responses were comparable to those observed in non-pregnant subjects [54,55]. By studying the evolution of HCV quasispecies throughout pregnancy, we also obtained evidence of the presence of statistically significant selective immune pressure exerted on specific regions of the E2 envelope protein during the third trimester of pregnancy, including E2 hypervariable regions 1 and 3 (HVR1 and HVR3) [56]. The foetus is also equipped with its own developing yet already active immune system that can modulate the manner in which the mother responds to her antigenic environment [21]. Indeed, significant HIV-specific cytotoxic T lymphocyte (CTL) responses are detected in uninfected children born to HIV-infected mothers [57,58], suggesting that CTL priming occurred in these subjects and that the resulting responses conferred a degree of protection against HIV infection. T cells are already detectable during gestational week 10 and mature and functional CD8^+^ T cell responses to congenitally-acquired human cytomegalovirus (CMV) can be detected as early as 28 weeks of gestation, suggesting that the human foetal immune system is capable of effector responses [59,60,61]. Thus, although no HCV-specific T cell responses were observed in umbilical cord blood samples obtained from children born to HCV-infected mothers [62], and although a footprint of foetal immune responses on maternal circulating viral populations has to our knowledge never been evidenced, antiviral immune responses in pregnancy should be considered as the sum of the responses that originate from the maternal immune system and from that of the foetus, thereby representing a unique immunologic landscape that may be modulated but is certainly not strictly suppressed [21].

Parturition, the final phase of pregnancy, is characterized by a pro-inflammatory environment that promotes uterine contractions, rejection of the placenta, and delivery of the newborn child [21]. It was shown that a state of broad immune activation develops in pregnant women at or near delivery [63]. During the course of a normal pregnancy, levels of neopterin, a blood serum marker of immune activation, rise before delivery and decline 2 months post-partum, while levels of soluble CD8 (sCD8), another marker of immune activation, increase at delivery and peak at 2 months post-partum. In addition, 6 to 8 weeks post-partum, the frequency of CD4^+^ CD25^+^ Treg cells in the peripheral circulation rapidly declines to pre-pregnancy levels [47]. Interestingly, 3 months after delivery, serum HCV RNA levels decrease and serum ALT levels increase to reach values similar to those found before pregnancy [15,30]. One can hypothesize that the loss of pregnancy-associated immune tolerance, concomitant with the pro-inflammatory environment associated with parturition, leads to a surge in maternal HCV-specific cell-mediated immune responses that results in a better control of HCV replication. Honegger *et al.* [55] reported that the decline in HCV viral load observed between the third trimester and the post-partum period was associated with an important (100%) increase in HCV-specific CD4^+^ and CD8^+^ T cell responses, as measured using IFN-γ ELISpot and overlapping peptide panels. In addition, the breadth of the average T-cell response was also improved post-partum, targeting twice as many HCV peptide pools than during the third trimester [55]. These results are consistent with a model in which the post-partum rebound in liver inflammation and decline in HCV viral load could stem from the rekindling of HCV-specific T cell responses and ensuing hepatic pathology resulting from destruction of infected hepatocellular tissue. Finally, Hattori *et al.* reported that 2 of 22 pregnant women with chronic HCV infection experienced «spontaneous» resolution of HCV viremia following parturition [64]. This rate of clearance was significantly greater than that observed in a non-pregnant control group (n = 120). In addition, HCV core protein levels at 3 months post-partum were much lower among patient who cleared HCV viremia than in those who had persistently detectable HCV RNA levels [64]. Overall, these findings testify to a remarkable degree of reconstitution of HCV-specific cell-mediated immune responses following parturition in HCV-infected women. 

## 3. Transmission of HCV from Mother to Child

In developed countries, MTCT is the leading cause of pediatric HCV infection. Multiple host factors were shown to increase the risk of HCV MTCT, including amniocentesis, prolonged rupture of membranes, and elevated HCV viral load in the mother. Indeed, perinatal HCV transmission is almost restricted to women with detectable HCV RNA in the peripheral blood and MTCT rarely occurs if the maternal viral load remains below 1 × 10^5^ HCV RNA copies/mL plasma [10,13,14,15,16,65,66]. However, there is a broad overlap in the levels of plasma HCV RNA between transmitting and non-transmitting mothers [66]. 

Standard of care treatment for chronic HCV infection is a combination of pegylated interferon alfa (IFN-α) and ribavirin [67,68]. Recently-developed DAAs such as telaprevir and boceprevir [69,70] can be added to this backbone to enhance the rate of sustained virological response (SVR). MTCT of HIV-1 can be efficiently prevented by antiretroviral prophylaxis and programmed cesarean section [71,72]. However, in the case of hepatitis C, ribavirin is contraindicated for use in pregnancy (FDA Pregnancy Category X), as it was shown to be teratogenic in multiple animal species [73,74,75]. IFN-α, a Pregnancy Category C drug, does not appear to exert overtly adverse effects on the foetus, but clinical experience is limited [76,77]. Furthermore, use of IFN-α in children was associated with abnormal neurologic examinations [78]. Finally, whether programmed cesarean section is effective in preventing MTCT of HCV is unclear based on published evidence [79,80,81,82,83]. This has interesting implications regarding the timing and actual mechanisms that are involved in mother-to-child HCV transmission, which might take place earlier during gestation as compared with HIV-1 transmission. Indeed, the detection of HCV viral RNA in some infants in the first 3 days of life suggests that early *in utero* infection is possible [65,84]. In fact, more than one third of infected children acquire HCV by this route of transmission and up to one-half by late intrauterine and intra-partum transmission, as evidenced by detectable HCV RNA levels several weeks after birth [65] and by the presence of HCV variants that are not contemporaneous with the maternal quasispecies at birth [85]. 

Two studies have shown that high ALT levels in the year preceding pregnancy and at delivery are associated with higher rate of MTCT, suggesting that maternal development of hepatocellular injury is a potential risk factor for HCV vertical transmission [86,87]. Moreover, HCV infection and signs of viral replication in maternal peripheral blood mononuclear cells (PBMC) increases the risk of vertical transmission [88,89]. In contrast, breastfeeding, HCV genotype and mode of delivery were not associated with MTCT of HCV [66]. 

**Figure 1:**
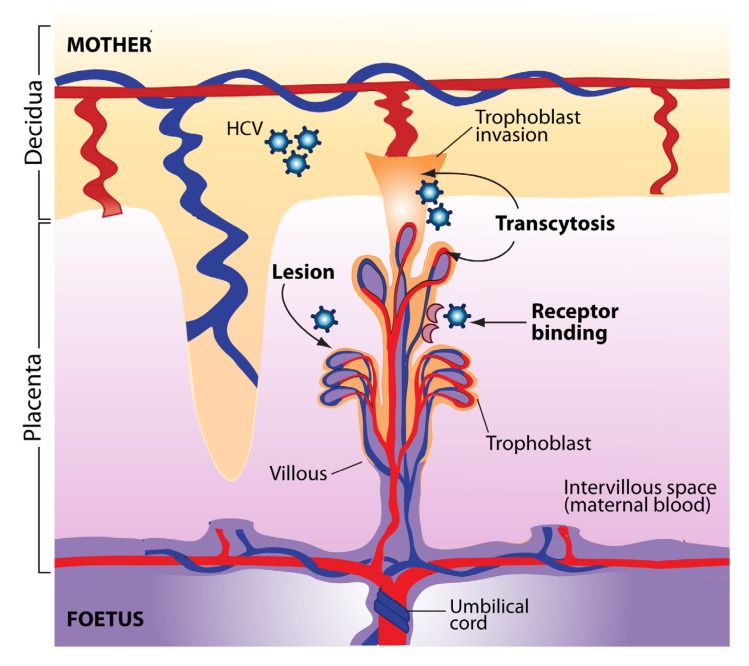
Potential pathways of hepatitis C virus (HCV) transmission to the foetus at the uterine-placental interface. The structural units of the placenta are the chorionic villi that float in maternal blood (intervillous space). Villi are composed of a stromal core with blood vessels, surrounded by cytotrophoblast progenitor cells. As a part of their differentiation program, extravillous cytotrophoblasts join a column at the tips of the anchoring villi and invade the uterine wall. Syncytiotrophoblasts that cover floating villi mediate exchanges of substances and passive transfer of IgG from maternal blood to the foetus. HCV transmission to the foetus could occur through viral transcytosis across trophoblast cells, could be mediated by HCV receptors expressed at the surface of placental cells, or could result from direct or indirect injury that compromise the integrity of the placental barrier.

Most importantly, multiple studies have shown that the rate of MTCT of HCV is increased 3-4-fold when the mother is coinfected with HIV-1, the etiologic agent of AIDS [16,79,90,91]. In a meta-analysis of 10 studies, it was shown that maternal coinfection with HCV and HIV-1 increases the odds of vertical HCV transmission by 90% compared with maternal HCV infection alone [92]. Some studies suggest that this increase occurs mainly in the context of co-transmission [93]. How HIV-1 infection enhances the rate of HCV transmission is unclear. This could result from higher HCV viral load in HIV-infected transmitters, though this was not consistently observed [8,12]. HIV-1 infection facilitates HCV entry and replication in PBMC, which constitutes a risk factor for vertical HCV transmission [88,94]. PBMC could act as a vehicle for the virus and transfer it to target cells in the new host, or produce HCV-containing exosomes that can by themselves enter the foetal bloodstream and lead to vertical transmission [89]. Another possibility is that HIV-induced immune suppression interferes with HCV-specific innate and/or humoral and/or cell-mediated immunity at the maternofoetal interface. As HIV-1 infects trophoblasts [95], yet another possibility is that this leads to a compromise in the integrity of the placental barrier, leading to transport of HCV-infected cells and/or free virus in the foetal circulation. Alternatively, HIV-associated chorioamnionitis could also lead to the generation of placental microtransfusions through which HCV infection can be transmitted to the foetus [96] (Figure 1).

Protection from MTCT requires the coordination of multiple components of the immune response, including cell migration for surveillance and recognition [21]. The decidua, placenta and umbilical cord provide a direct connection and regulate the exchanges between the mother and the foetus: in addition to a variety of cell-free substances, foetal and maternal cells transit though the placenta in both directions. Maternal DNA can be detected in foetal lymph nodes, resulting in a maternal microchimerism [97]. Maternal cells in the foetus promote the generation of foetal Treg cells and promote foetomaternal tolerance [98]. It is suggested that HCV transmission occurs directly through the placenta, as the amniotic fluid does not show evidence of accumulation of HCV RNA [99]. The uterine decidua contains large amounts of immune cells, including macrophages, T lymphocytes and natural killer (NK) cells [21]. The placenta is also able to recognize and respond to microbial pathogens. It is made up of chorionic villi and a layer of trophoblasts (syncytiotrophoblasts and cytotrophoblasts) [100]. Syncytiotrophoblasts make up the maternofoetal interface through which the exchange of substrates occurs via transcytosis. Infection of the placenta and of the foetus depends on the relative permissiveness of these cells to different pathogens. Placental cells produce multiple cytokines, chemokines and hormones. For instance, the production of IFN-β and secretory leukocyte protease inhibitor (SLPI) by trophoblasts following TLR-3 stimulation in response to a viral infection is thought to interfere with MTCT [21]. Transmission of HBV from mother to child can occur early in pregnancy via viral transcytosis across the undifferentiated trophoblasts layer [101]. This was also shown for HIV-1 [102]. Babik *et al.* estimated that 1 × 10^13^-1 × 10^14^ HCV virions reach the placental bed during gestation, making highly probable that some particles would cross the placenta even if transmission is inefficient [62]. Thus, foetal exposure to HCV would take place more frequently than actual *in utero* transmission, perhaps even altering the balance between suppressive and pro-inflammatory responses in the process [62]. Low levels of chronic T cell activation may be beneficial in promoting T cell function, and IFN-γ produced in response to *in utero* exposure might play a role in the protection against viral infection, as shown in the case of HIV-1 [103,104]. The mechanisms by which HCV can cross the placenta and the pathologic consequences of placental HCV infection are not clear at the present time. There are several potential sites in the placenta that could allow the passage of free virions or cell-associated virus [105]. Since expression of many HCV receptors and entry cofactors have been detected in the placenta, including claudin-1, occludin, SR-B1, LDLr, and DC-SIGN, it is possible that HCV can directly infect placental cells [106,107,108], something that was recently demonstrated *in vitro* using human cytotrophoblasts [109] (Figure 1).

In an elegant study, Hutardo *et al.* performed a characterization of innate immune profiles in samples of decidua, placenta, and umbilical cord blood obtained from pregnant women with chronic HCV infection and their infants, as well as healthy mother-infant pairs [110]. Their results revealed the existence of a gradient in the relative frequencies of natural killer (NK) T cells and γδ T cells, both of these cell types being more frequent in placenta than cord blood. NK T cell and γδ T cell frequencies were even higher in placenta from HCV-infected women. Production of cytokines by NK cells was robust and cytotoxicity mediated by NK T cells was also increased in HCV-exposed placenta [110]. Considering that NK cells [111,112] and NK T cells [113] have been shown to play an important role in the clearance of acute HCV infection, these results provide a potential mechanism by which the placenta could prevent MTCT. Studies that analyzed the quasispecies profile based on HVR1 in HCV-infected infants before seroconversion revealed a limited diversity of viral variants [114,115,116]. This suggests that vertical transmission of HCV may involve only a restricted number of viral variants. Moreover, it has been shown that in the context of coinfection with HCV and HIV-1, maternal HCV-specific neutralizing antibodies do not contribute to the prevention of HCV vertical transmission [117]. However, this investigation was restricted to women who were coinfected with HIV-1, who generally have low titres of neutralizing antibodies. A recent longitudinal study of 12 HCV-infected children during their first year of life revealed the presence of neutralizing antibodies in 3 mothers, strongly suggesting that they were not sufficient for the prevention of MTCT [118]. 

## 4. Pathogenesis of Hepatitis C in Childhood

Considering the overall prevalence of HCV infection and the number of women of reproductive age, it has been suggested in 2001 that between 10,000 and 60,000 newborns were infected with HCV each year via vertical transmission [119]. During the first year of life, anti-HCV serologic positivity may represent passively transferred maternal antibodies, there may be intermittent detection of HCV RNA, and serum ALT may not be elevated. CDC guidelines recommend testing for anti-HCV antibodies in children born to HCV infected mothers after 12 months of age, as passively-acquired maternal antibodies will have generally waned by that time. However, if earlier testing is required, nucleic acid-based testing for HCV RNA is recommended 1 to 2 months after birth [120,121].

Comparatively little is known about the natural history of hepatitis C in children. It was shown that perinatally-acquired HCV infection becomes chronic in approximately 80% of cases [91,122,123,124], a rate similar to that observed in adults [125], but higher than that reported in children who were infected following transfusion with HCV-contaminated blood products [126]. If clearance occurs, it tends to take place early in infection. Younger age and normal ALT levels have been associated with spontaneous clearance in a group of 157 children with transfusion-acquired and non-transfusion-acquired hepatitis C [124]. Spontaneous clearance was also recently associated with the presence of positive IFN-γ responses directed against structural and non-structural recombinant HCV antigens [127]. Chronic HCV infection appears to have a different clinical course in children as compared to adults. First, pediatric HCV infection is associated with minimal or mild liver disease, and advanced liver damage is not a common finding [128,129,130]. A broad range of ALT levels have been observed during the first year of life, with some infants exhibiting levels consistent with acute hepatitis and others showing normal or almost normal levels [114,122,129]. The association between evolution of the viral quasispecies and the clinical course of pediatric HCV infection has been examined in different studies with contrasting results. Farci *et al.* showed that the pattern of viral evolution correlated with ALT profiles but was independent of HCV viral load [114]. Biochemical evidence of hepatic injury was associated with mono- or oligoclonal populations of viral variants, whereas absence of or mild liver damage was temporally associated with the emergence of heterogeneous viral quasispecies and with the appearance of anti-HCV antibodies [114]. The authors hypothesized that heightened ALT levels may reflect the action of nascent HCV-specific cell-mediated immune responses, while normal ALT levels would result from an absence or impairment of HCV-specific cytotoxic T cells (anergy; clonal exhaustion; CTL escape) and from the presence of robust antibody responses capable of exerting strong selective pressures, leading to the diversification of the HCV variant spectrum. While results from other groups support these observations [131], other studies reported a gradual diversification of HCV quasispecies independent of serum ALT levels [115]. Protracted evolution of the variant profile was also observed in children who acquired both HCV and HIV-1 via MTCT [132,133], in HCV-infected children suffering from X-linked agammaglobulinemia [134], and in an anecdotal case of persistently seronegative, perinatally-acquired HCV infection [135]. In adults, chronic HCV infection may lead to cirrhosis and hepatocellular carcinoma in 10 to 20% of patients [136]. No studies have yet examined the incidence of cirrhosis and hepatocellular carcinoma in adults who acquired hepatitis C as a result of MTCT. Finally, treatment modalities that were initially restricted to adult subjects (*i.e.,* pegylated IFN-α combined with ribavirin) are now recommended for the treatment of chronic hepatitis C in children 3–17 years of age [137,138,139], leading to high rates of SVR in these young patients. Therapeutic options will probably be expanding in the future. For example, the DAA boceprevir is currently in phase I clinical trial in children 3–17 years of age infected with HCV genotype 1 [140].

## 5. Conclusions

HCV infection affects a large number of women of reproductive age worldwide, and transmission of HCV from mother to child remains a serious public health problem. The pathogenesis of hepatitis C during pregnancy is in all likelihood strongly affected by immunological changes associated with gestation and maternofoetal tolerance. This leads to heightened HCV viral load in the third trimester of pregnancy, a situation which may provide the virus with improved opportunities to disseminate. The rebound of hepatocellular damage observed following parturition could be associated with a rekindling of HCV-specific cell-mediated immune responses that are attempting to bring viral replication under control. Interestingly, this period might in fact represent a strategic timeframe to initiate antiviral treatment with pegylated IFN-α, ribavirin, and DAAs, with the objective of achieving superior rates of SVR.

At the same time, in spite of the seemingly overwhelming exposure of the foetus to HCV virions, antigens, and maternal HCV-infected PBMCs, the rate of MTCT remains surprisingly modest when compared to HIV-1 or CMV. This underlines the uncanny efficacy of the restriction mechanisms that not only physically isolate the foetus from contact with HCV, but also provide innate and adaptive defences against viral infection. The characterization of the precise nature of these mechanisms should be the focus of further research, as they hold the keys to a novel understanding of host-pathogen interactions, and, potentially, to the development of new antiviral approaches.
